# A Novel Immune-Related ceRNA Network and Relative Potential Therapeutic Drug Prediction in ccRCC

**DOI:** 10.3389/fgene.2021.755706

**Published:** 2022-01-25

**Authors:** Weiquan Li, Xiangui Meng, Hongwei Yuan, Wen Xiao, Xiaoping Zhang

**Affiliations:** ^1^ Department of Urology, Union Hospital, Tongji Medical College, Huazhong University of Science and Technology, Wuhan, China; ^2^ Shenzhen Huazhong University of Science and Technology Research Institute, Shenzhen, China; ^3^ Institute of Urology, Tongji Medical College, Huazhong University of Science and Technology, Wuhan, China

**Keywords:** ccRCC, ceRNA, drug prediction, bioinformatics analysis, immune-related

## Abstract

Renal cell carcinoma (RCC) is the third common solid tumor in the urinary system with a high distant metastasis rate. The five-year survival rate of RCC has reached 75%, benefiting from the emergence and update of multiple treatments, while its pathogenesis and prognostic markers are still unclear. In this study, we committed to explore a prognostic ceRNA network that could participate in the development of RCC and had not been studied yet. We screened nine immune-related hub genes (AGER, HAMP, LAT, LTB4R, NR3C2, SEMA3D, SEMA3G, SLC11A1, and VAV3) using data of The Cancer Genome Atlas Kidney Clear Cell Carcinoma database (TCGA-KIRC) through survival analysis and the cox proportional hazard model. Next, we successfully constructed a ceRNA network of two mRNA (NR3C2 and VAV3), miRNA (hsa-miR-186-5p), and lncRNA (NNT-AS1) for ccRCC based on numerous online bioinformatics tools and Cytoscape. Finally, we predicted five potential drugs (clemizole, pentolonium, dioxybenzone, Prestwick-691, and metoprolol) based on the above results.

## Introduction

Renal cell carcinoma (RCC) is one of the most common tumors all over the world, accounting for 5% of all new cancer cases in men and 3% in women (Siegel et al., 2021). With the increasing development of treatment approaches, the five-year survival rate has reached 75% for RCC in all stages and 93% for patients with localized lesions (Posadas et al., 2017). Clear cell renal cell carcinoma (ccRCC) accounts for more than 80% of RCC in different pathological subtypes and is the main concerned type (Capitanio and Montorsi, 2016). It is known that more than 30% cases are accidentally discovered during daily health checkups, instead of the typical symptoms. It is necessary to find effective biomarkers for ccRCC patients.

Nowadays, there are numerous research studies to figure out the development of ccRCC and how to treat it. In addition to the classic and abundant lipid-related treatments researched (Xiao et al., 2019), studies on immune-related treatments are also in full swing (Sharma and Allison, 2015; Barata and Rini, 2017; Wei et al., 2018). It is meaningful to seek out new immune targets for therapy of ccRCC patients.

ceRNA (competing endogenous RNA) hypothesis indicates that some molecules, like long non-coding RNA (lncRNA), can compete with the same microRNA (miRNA) response elements, thereby affecting gene expression (Salmena et al., 2011). Various studies proved that the ceRNA network could affect cancer cell proliferation and migration and serve as suitable therapeutic targets (Martens-Uzunova et al., 2014; Qu et al., 2016; Zhai et al., 2017; Zhai et al., 2019). However, the immune-associated ceRNA network was little studied in ccRCC.

In this study, we constructed a new immune-related network and validated the prognostic value of each element of the network. The potential signaling ways were analyzed by online bioinformatic tools. Several therapeutic drugs for ccRCC patients were also predicted based on the three target genes we screened.

## Materials and Methods

### Data Collection and Differentially Expressed Gene Screening

The mRNA-seq data, lncRNA-seq data, and clinical information of ccRCC in The Cancer Genome Atlas Kidney Clear Cell Carcinoma database (TCGA-KIRC) were downloaded from the Xena Functional Genomics Explorer (Xiao et al., 2020). All 1793 immune-related genes were collected from ImmPort (https://www.immport.org) (Bhattacharya et al., 2018). There were 2483 rows of data that were included in the file we downloaded from ImmPort; then, the duplicate genes were eliminated and 1793 genes were left. We screened the differentially expressed genes (DEGs) of KIRC using the “limma” package with parameters of adjusted *p*-value < 0.05 and log_2_|FC| > 1. Then, the intersection of DEGs and 1793 immune-related genes were obtained using a Venn diagram.

### Functional Enrichment, Interaction Network Analysis, and Hub Gene Identification

The Database for Annotation, Visualization, and Integrated Discovery (DAVID) was used to obtain the functional enrichment results of 586 DEGs. We selected the top15 enrichment results of the biological process (BP), cellular component (CC), and molecular function (MF) to present. The top 20 signaling ways of Kyoto Encyclopedia of Genes and Genomes (KEGG) results were shown. *p*-value < 0.05 was considered significant. The protein-protein interaction (PPI) network of all DEGs was pictured by Cytoscape (v.3.6.1) using the “string” plugin. The confidence score was set as 0.40. Then, the PPI network was further analyzed by Cytoscape using the “MCODE” plugin to figure out the important modules. The top five modules were finally selected. Next, the “CytoHubba” plugin was conducted to screen the hub genes from all 586 DEGs with the criteria of degree >10.

### Survival Analysis and Multivariate Cox Regression Analysis

Patients of ccRCC were divided into high-expression groups and low-expression groups according to the median of each gene expression. The “survival” package and R software were used to evaluate the prognostic value of the hub genes. GEPIA was utilized to verify the overall survival (OS) prognosis of the above hub genes (Tang et al., 2017). Then, multivariate cox proportional hazard regression analysis was conducted by SPSS (version 23.0). The value of *p* < 0.05 was significant. There were nine genes (AGER, HAMP, LAT, LTB4R, NR3C2, SEMA3D, SEMA3G, SLC11A1, and VAV3) finally screened after the above three steps. The Metascape database was used for GO enrichment analysis for hub genes (Zhou et al., 2019).

### Prediction of miRNA and lncRNA and Construction of ceRNA

Starbase (Yang et al., 2011) and miRTarbase (Hsu et al., 2011) databases were used to predict miRNA reversely based on nine genes. The prediction results were merged and LAT was removed because there was no miRNA predicted. Cytoscape and plugin “CytoHubba” were conducted to obtain the top 15 miRNA according to the score of degree. The expression of miRNA was explored by UALCAN (Chandrashekar et al., 2017). The survival analysis of miRNA was identified by OncoLnc (http://www.oncolnc.org/). Next, LncBase (Paraskevopoulou et al., 2013) and Starbase databases were used to predict lncRNA based on miRNA, both of which had different expressions and were related to patients’ survival. There were 29 lncRNA in the intersection of prediction results, which were screened. Graphpad prism (v 8.2.1) was used to analyze the differential expression of lncRNA through an unpaired t-test, OncoLnc was used for survival analysis, and SPSS was used to conduct multivariate cox proportional hazard regression analysis. Finally, we constructed a new immune-related ceRNA network including NNT-AS1 (lncRNA), hsa-miR-186-5p (miRNA), NR3C2, and VAV3.

### Gene Set Enrichment Analysis and Screening of Potential Drugs

LinkedOmics (Vasaikar et al., 2018) was utilized to conduct the Gene set enrichment analysis (GSEA) of two genes in the ceRNA network (NR3C2 and VAV3). Connectivity Map (Cmap) (Lamb, 2007) was a common small-molecular drug-prediction database, so we predicted potential therapeutic chemicals for ccRCC through three genes (NR3C2, VAV3, and HAMP) associated with miR-186-5p. PubChem (Kim et al., 2018) was used to find their structure and other information.

### Immune Infiltration Analysis of VAV3 and NR3C2

In order to explore the relationship between immune-infiltration level and the two immune genes, we used CIBERSORT (Chen et al., 2018; Kong et al., 2020; Meng et al., 2021a) (https://cibersort.stanford.edu/) to obtain the immune infiltration level of TCGA-KIRC data. The algorithm was run using LM22 signature and 1,000 permutations. The 530 samples of TCGA-KIRC were divided into two groups according to the median of VAV3 or NR3C2. Then, R software and the “ggplot2” package were used to further present the immune cell infiltration level difference between two groups. The Wilcoxon test was used to calculate the *p* value. *p* value <0.05 was considered significantly.

### Validation of Quantitative Real-Time Polymerase Chain Reaction

Paired normal and cancer tissues of ccRCC were obtained from Department of Urology, Union Hospital, Tongji Medical College, Wuhan, China, with the approval of the Ethics Committee of Huazhong University of Science and Technology. According to the instructions, we performed RNA Isolation and Real-time PCR analysis with SYBR Green mix (Thermo, Massachusetts, USA). Primers of NNT-AS1 and hsa-miR-186-5p were obtained from RiboBio (Guangzhou, China). Primers of VAV3, NR3C2, and normalized gene GAPDH were obtained from Sangon Biotech (Shanghai):

GAPDH

Forward 5′‐GAG​TCA​ACG​GAT​TTG​GTC​GT‐3′

Reverse 5′‐GAC​AAG​CTT​CCC​GTT​CTC​AG‐3′

VAV3

Forward 5′‐AGA​GAA​ACG​GAC​CAA​TGG​ACT‐3′

Reverse 5′‐GGT​GGT​GTT​CCA​GAA​TAG​TTC​C‐3′

NR3C2

Forward 5′‐GAA​AGA​CGG​TGG​GGT​CAA​GTT‐3′

Reverse 5′‐ACC​GGA​AAC​ACA​GCT​TAC​GTT‐3′

## Results

### Determination of Immune-Related DEGs

**FIGURE 1:**
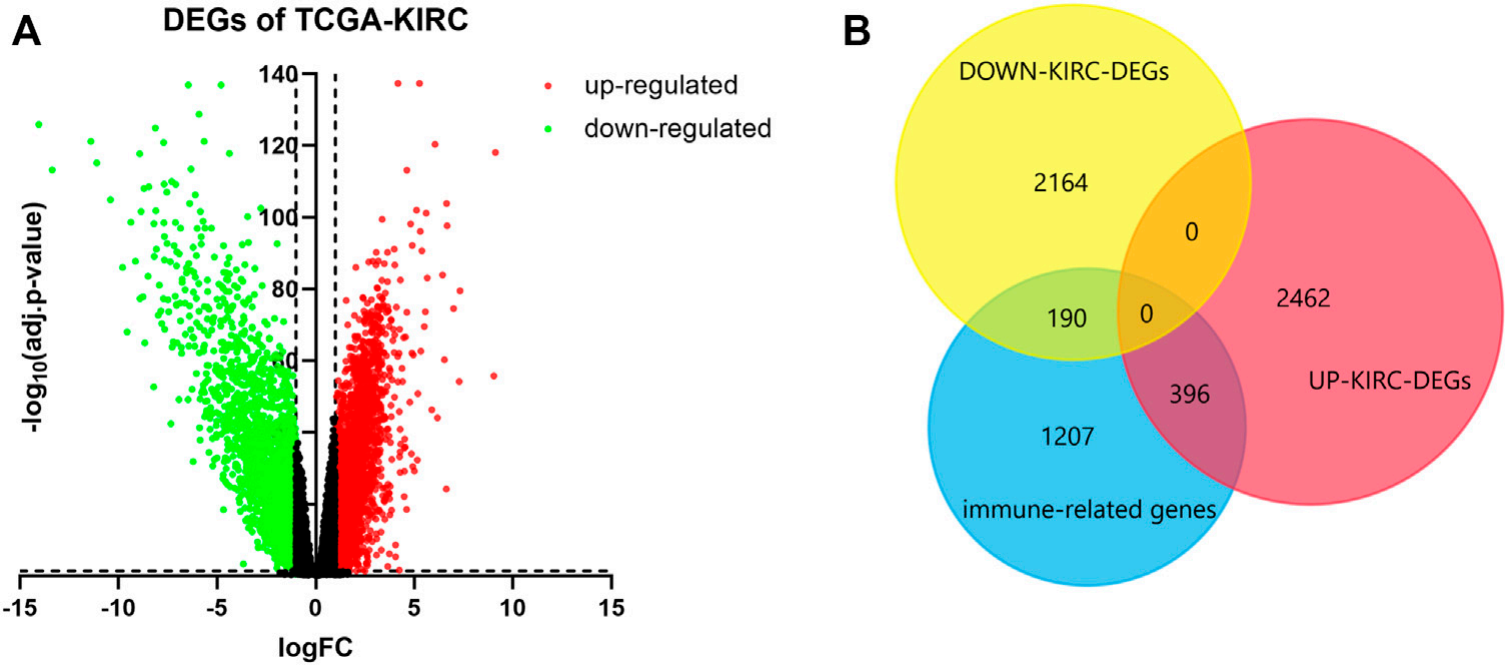
Determination of immune-related DEGs. **(A)** Volcano of DEGs using data from TCGA-KIRC. Adj.*p*-value < 0.05; log_2_|FC| > 1. Red points represent upregulated genes, and green points represent downregulated genes. **(B)** Venn plot of DEGs and immune genes.

We screened 2858 upregulated DEGs and 2354 downregulated DEGs in the TCGA-KIRC database, setting the parameters of *p* < 0.05 and log_2_|FC| >1 (Figure 1A). Taking the intersection of DEGs and 1793 immune-related genes, 396 upregulated immune genes and 190 downregulated immune genes were finally determined and used for the following analysis (Figure 1B).

### Functional Enrichment Analysis, Construction of PPI, and Module Clustering of Immune-Associated DEGs

**FIGURE 2:**
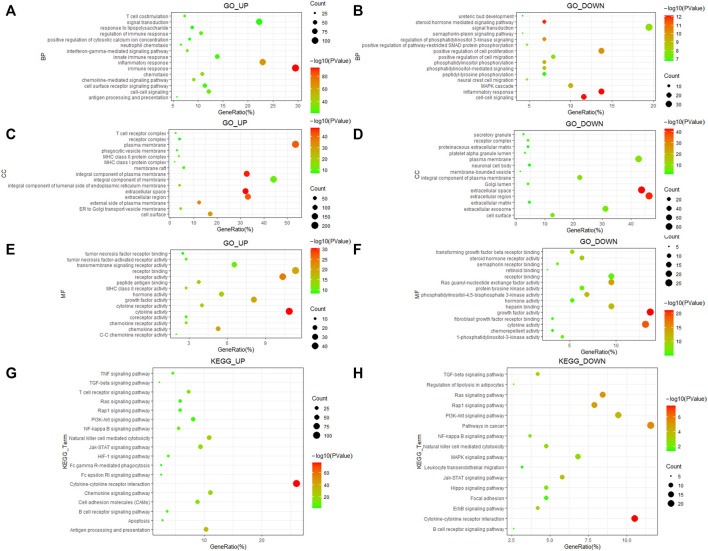
GO and KEGG enrichment of 586 immune-related DEGs. **(A,B)** Biological process of DEGs. **(C,D)** Cellular component of DEGs. **(E,F)** Molecular function of DEGs. **(G,H)** KEGG pathways enrichment results of DEGs.

To determine the biological functions and potential signaling ways of DEGs, we used the DAVID database to perform GO and KEGG analysis of them. We selected the top 15 terms of GO and the top 20 terms of KEGG since there too many enrichment results. The results indicated that upregulated DEGs were enriched in biological processes (BP) of T-cell costimulation and interferon-gamma mediated signaling ways, while downregulated DEGs participated in cell proliferation and migration (Figures 2A,B). Cellular component (CC) enrichment results showed that both upregulated and downregulated DEGs were involved in the formation of various membrane and receptor complexes (Figures 2C,D). Molecular function (MF) results suggested that upregulated DEGs were associated with the tumor necrosis factor receptor, tumor necrosis factor-activated receptor activity, and so on (Figure 2E). Downregulated DEGs were related to transforming growth factor-beta receptor binding, fibroblast growth factor receptor binding, and so on (Figure 2F). KEGG enrichment indicated that both groups participate in various important signaling ways in ccRCC development (Figures 2G,H).

**FIGURE 3:**
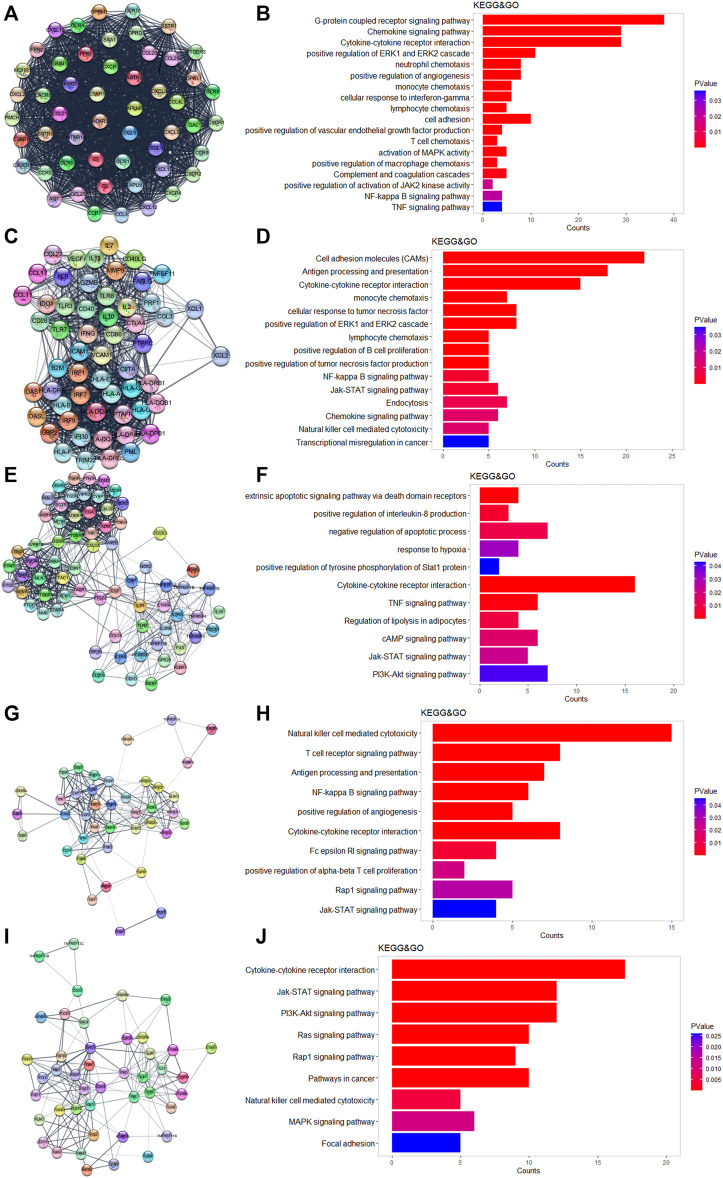
Results of MCODE in DEGs. **(A,C,E,H,I)** Top five modules calculated by MCODE plugin. **(B,D,F,H,J)** GO and KEGG enrichment results of the five modules.

We used Cytoscape and the “string” plugin to construct the PPI network of all 586 DEGs. Then, “MCODE” plugin was utilized to divide the DEGs into several modules. We selected the top five modules for the following enrichment analysis. Genes in module 1 were associated with neutrophils, monocytes, lymphocyte chemotaxis, and positive regulation of angiogenesis (Figures 3A,B). Genes in module 2 were involved in cell adhesion molecules, positive regulation of B-cell proliferation, and natural killer cell-mediated cytotoxicity (Figures 3C,D). The TNF-signaling pathway and IL8 production were enriched in module 3 (Figures 3E,F). The NF-kappa B signaling pathway, Rap1 signaling pathway, and JAK-STAT signaling pathway were enriched in module 4 (Figures 3G,H). It was worth mentioning that the above three pathways were also enriched in the other four modules. DEGs in module 5 were related to the PI3-Akt signaling pathway and MAPK signaling pathway (Figures 3I,J).

### Determination of Hub Genes and Survival Analysis

**FIGURE 4:**
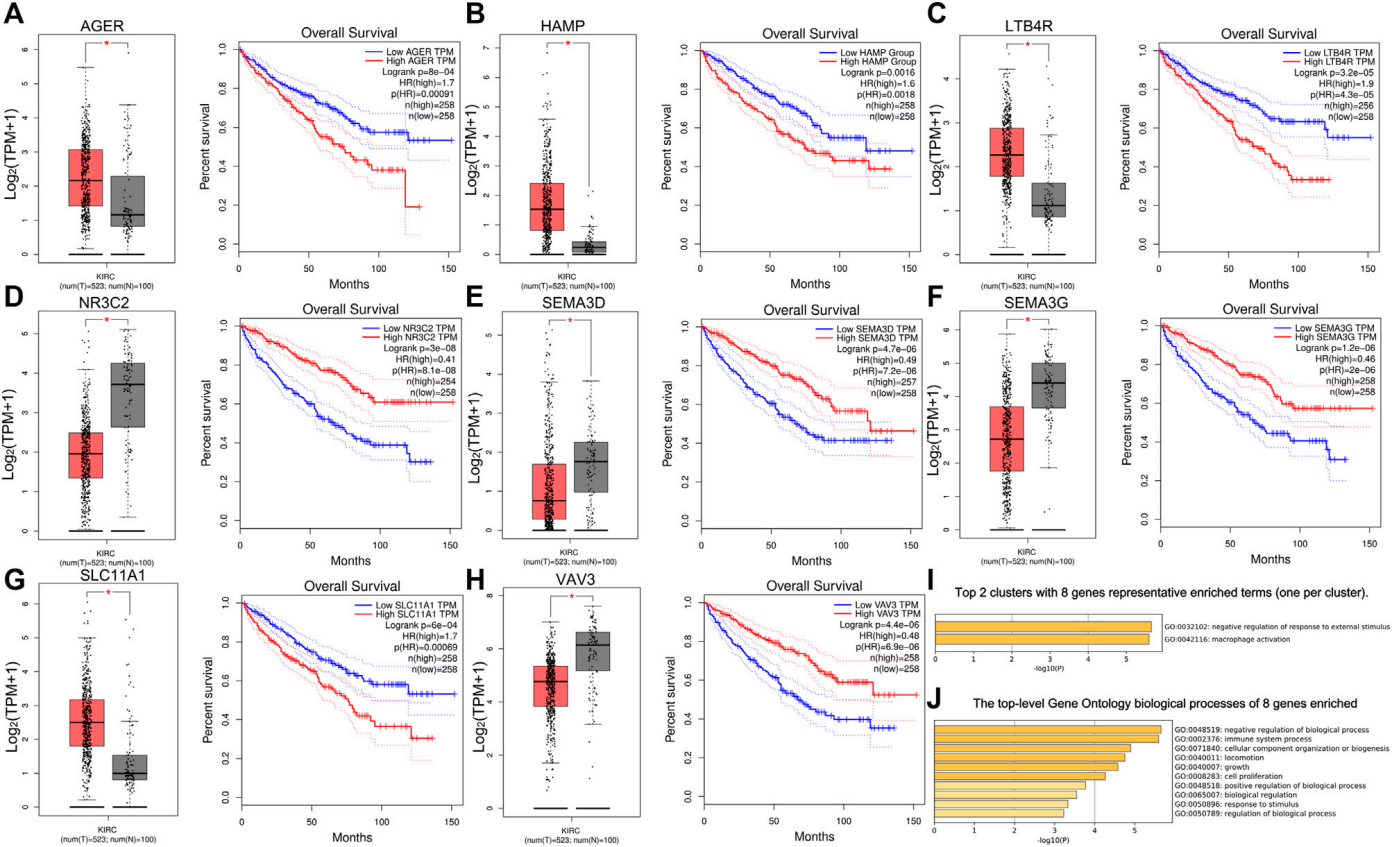
Survival analysis of eight hub genes. **(A–H)** Different expressions and overall survival analysis of eight hub genes based on GEPIA. AGER, HAMP, LTB4R, and SlC11A1 were highly expressed and acted as a risk factor in ccRCC. NR3C2, SEMA3D, SEMA3G, and VAV3 had lower expression levels in ccRCC and served as a protective factor. **(I)** Eight hub genes could be divided into two clusters. **(J)** Biological processes of eight genes enriched.

First, we used Cytoscape and “CytoHubba” plugin to initially screen hub genes. There were 449 genes with degree >10 selected. Then, the OS rate of these genes was evaluated through the “survival” package. GEPIA was an online website by which we verified the prognostic value of the above genes. Multivariate cox proportional hazard regression analyses were further determined by the hub genes (Table 1). In addition, there were nine genes (AGER, HAMP, LAT, LTB4R, NR3C2, SEMA3D, SEMA3G, SLC11A1, and VAV3) finally selected. However, since later we found that there were no miRNA prediction results for LAT, we decided to remove it (Figures 4A–H). AGER, HAMP, LTB4R, and SLC11A1 were risk factors for ccRCC, while NR3C2, SEMA3D, SEMA3G, and VAV3 were protective factors for ccRCC. We obtained the GO enrichment results of the eight hub genes using the meta scale database. Interestingly, there were two clusters in which one (AGER, HAMP, SLC11A1, and VAV3) participated in macrophage activation (Figure 4I), and the eight hub genes were associated with cell proliferation and some immune processes (Figure 4J).

**TABLE 1:** OS analysis of nine hub genes with a prognostic value.

Gene	KM *p*-value^a^	Cox *p*-value^b^	Hr	95% CI low	95% CI high
AGER	8.81E-07	<0.001	1.828	1.333	2.170
HAMP	2.30E-10	0.027	1.442	1.041	1.996
LAT	5.50E-05	0.001	1.740	1.269	2.385
LTB4R	2.36E-08	<0.001	2.031	1.470	2.806
NR3C2	5.30E-07	0.005	0.607	0.429	0.858
SEMA3D	0.001464462	<0.001	0.540	0.390	0.749
SEMA3G	4.02E-08	0.009	0.640	0.458	0.894
SLC11A1	1.15E-08	0.001	1.718	1.253	2.355
VAV3	4.53E-07	0.008	0.642	0.462	0.892

a The *p*-values of KM analysis were calculated by the “survival” package, while the *p*-values of GEPIA were not present here.

b The multivariate cox regression analysis were calculated by SPSS. Parameters were set as follows: “Age > 60” = 1, “Age ≤ 60” = 0; “male” = 1, “female” = 0; “T3+T4” = 1, “T1+T2+Tx” = 0; “N1” = 1, “N0+Nx” = 0; “M1” = 1, “M0+Mx” = 0; “G3+G4” = 1, “G1+G2+Gx” = 0; “high expression” = 1, “low expression” = 0. The expression levels of genes were divided into two groups according to the median.

### Construction of the ceRNA Network Based on the Prediction

**FIGURE 5:**
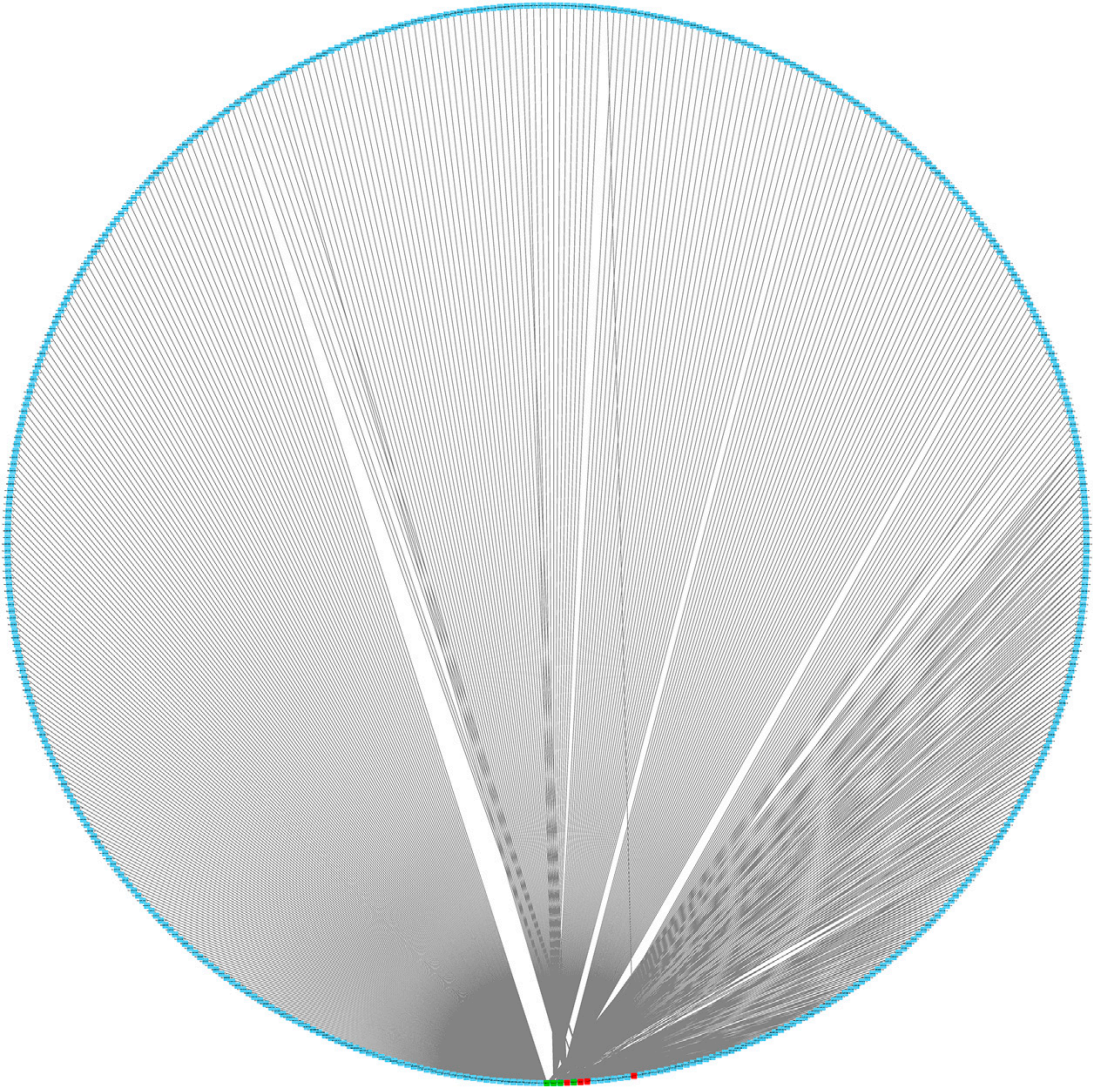
Interaction network of eight mRNA and reverse-predict miRNA. Red cubes represented highly expressed mRNA, green cubes represent lowly expressed mRNA, and blue cubes represent miRNA.

**FIGURE 6:**
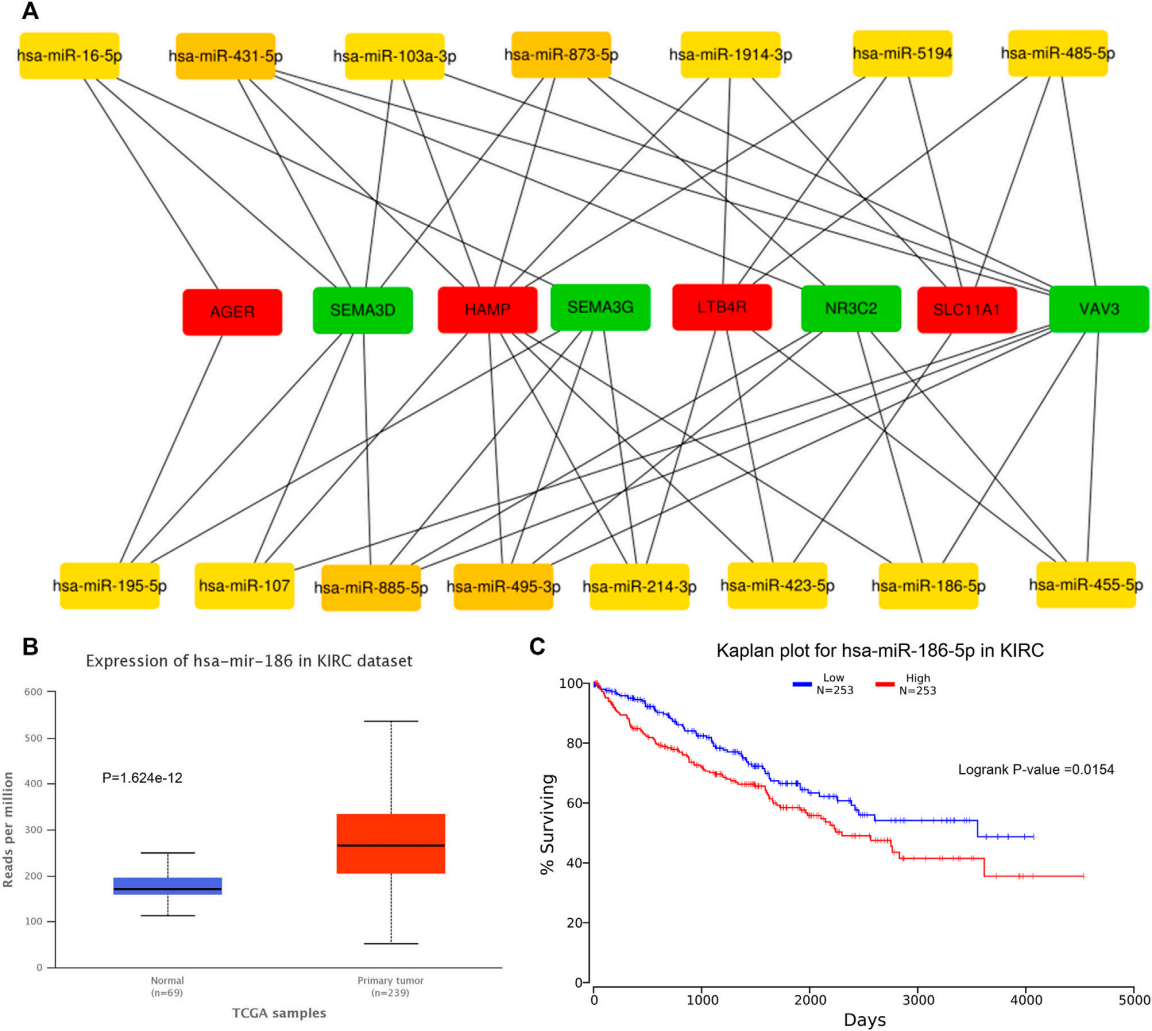
Prediction of hub miRNA. **(A)** Network of top 15 miRNA and 8 hub genes. **(B)** hsa-miR-186-5p expressed higher in the tumor tissue than in the normal tissue. *p* = 1.624e-12. **(C)** High hsa-miR-186-5p expression group showed poorer OS. *p* = 0.0154.

We predicted 673 miRNAs through Starbase and miRTarbase for all the eight hub genes (Figure 5). Then, Cytoscape and “cytoHubbe” plugin were used again to obtain the top 15 miRNAs associated with the hub genes (Figure 6A). Next, we explored the expression of miRNA using the UALCAN database and the prognostic value of them using the OncoLnc website. Then, hsa-miR-186-5p (alias, hsa-miR-186) was identified (Figures 6B,C). The related genes NR3C2 and VAV3 were negatively associated with hsa-miR-186-5p, which was consistent with the known mechanism of miRNA.

**FIGURE 7:**
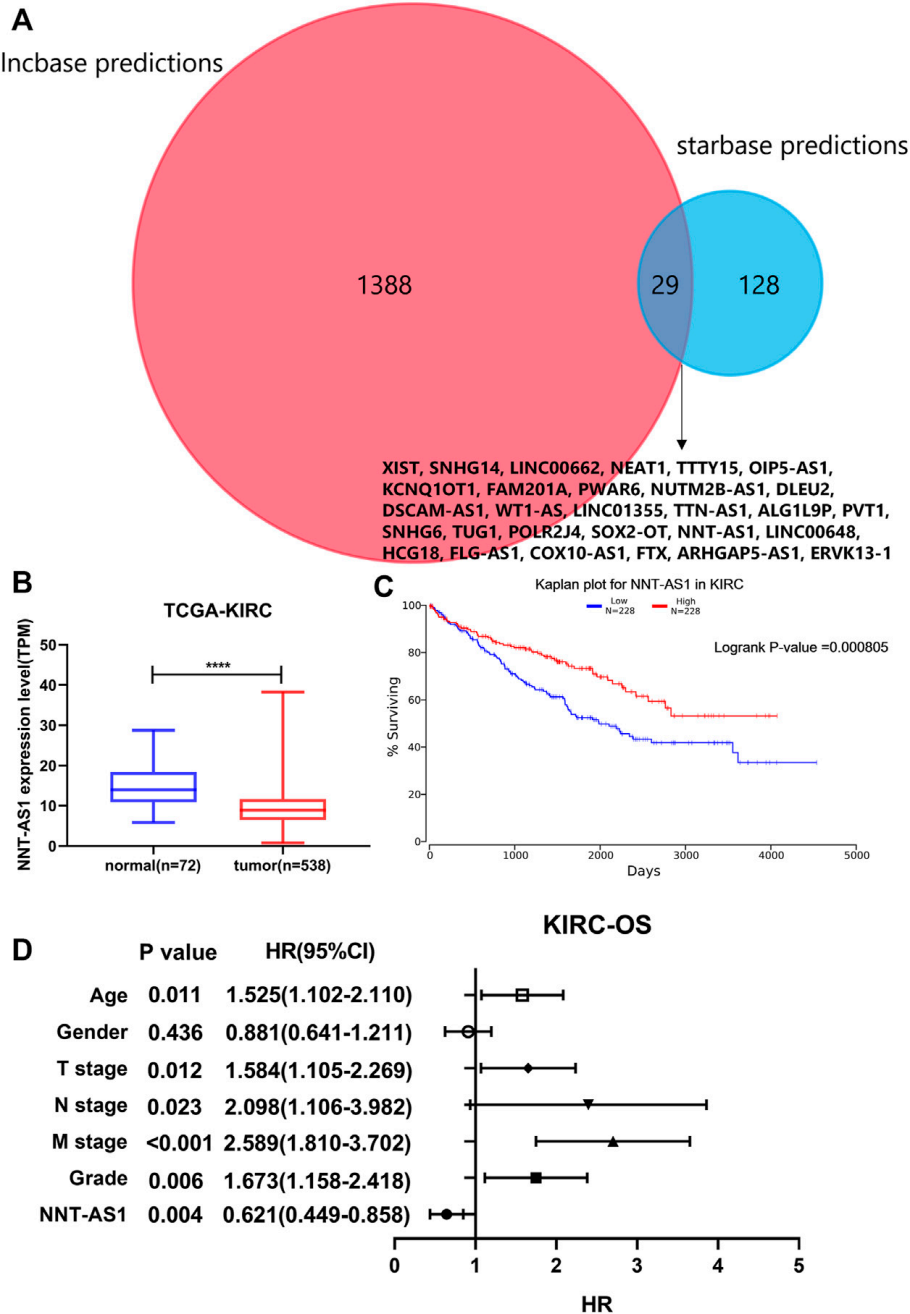
Prediction of lncRNA. **(A)** Intersection of predicted lncRNAs from two online databases. **(B)** NNT-AS1 expressed much lower in ccRCC samples. **(C)** Low expression of NNT-AS1 indicated a poorer survival rate. **(D)** Multivariate cox proportional hazard regression analysis of NNT-AS1 and other known risk factors. NNT-AS1 acted as a protective factor for ccRCC, *p* = 0.004.

As mentioned before, we predicted 1417 lncRNAs using Lncbase and 157 lncRNAs using Starbase for hsa-miR-186-5p. There were 29 lncRNAs in the intersection (Figure 7A). We checked their expression and conducted survival analysis and multivariate cox proportional hazard regression analyses to determine the final lncRNA. In addition, according to the ceRNA hypothesis, lncRNA should be negatively related to miRNA. Finally, NNT-AS1 was the only one suitable. NNT-AS1 had a much lower expression level in ccRCC tissues than in normal tissues (Figure 7B). The low expression group showed poorer survival (Figure 7C). The results of cox regression were presented in the forest plot (Figure 7D).

**FIGURE 8:**
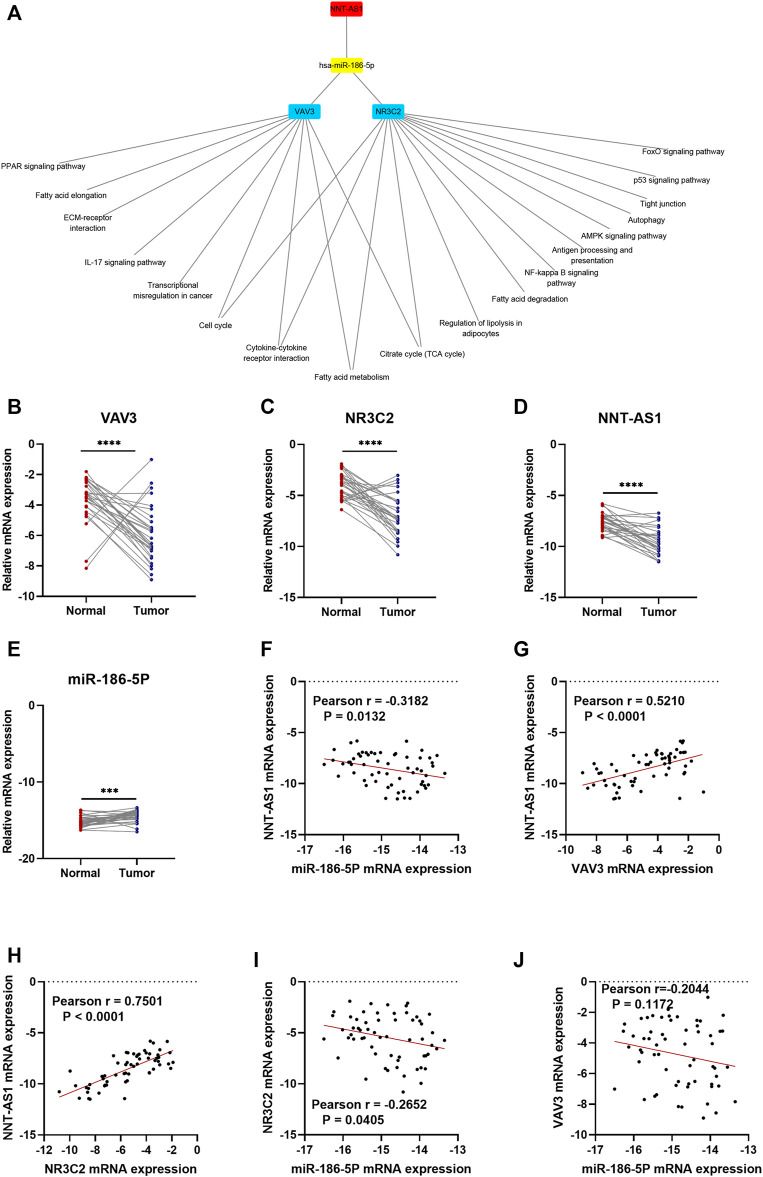
GSEA enrichment of NR3C2 and VAV3 separately. The CeRNA network associated expression level and correlation validation by qPCR. The expression levels of NNT-AS1. **(A)** hsa-miR-186-5p, **(B)** VAV3 **(C)**, and NR3C2 **(D)** were compared between normal tissues (*n* = 30) and ccRCC tissues (*n* = 30). The correlation analysis of the ceRNA network were conducted, and the R coefficients were calculated **(E–J)**.

As a result, a new ceRNA network (NNT-AS1—hsa-miR-186-5p—NR3C2/VAV3) was constructed by us. What is more, GSEA results of NR3C2 and VAV3 indicated that both genes were associated with the cell cycle, cytokine–cytokine receptor interactions, the fatty acid metabolism, and the citrate cycle (TCA cycle), which made great significance in the development of ccRCC (Figure 8A). To further verify the results from bioinformatics analysis, we performed qRT-PCR analysis on 30 pairs of clinical samples. As expected, the results showed that NNT-AS1, VAV3, and NR3C2 were remarkably downregulated in ccRCC compared to normal kidney tissues, while hsa-miR-186-5p was significantly highly expressed in ccRCC (Figures 8B–E). Moreover, the correlation analysis results further suggested that NNT-AS1 was negatively related to miR-186-5p (Figure 8F) but positively related to VAV3 and NR3C2 (Figures 8G,H). In addition, MiR-186-5p was negatively associated to VAV3 and NR3C2 (Figures 8I,J). In conclusion, the overall results were consistent with the hypothesis of the ceRNA network (NNT-AS1—hsa-miR-186-5p—NR3C2/VAV3).

### Immune Infiltration Analysis and Clinical Correlation of VAV3 and NR3C2

Since VAV3 and NR3C2 are both immune-related genes, we decided to further explore how these two genes influenced the immune infiltration of the ccRCC microenvironment using the CIBERSORT database. The results showed that the proportions of CD4^+^ memory resting T-cells, monocytes, M1, M2, resting DCs, and resting Mast cells were higher in VAV3high than in VAV3low groups. On the contrary, plasma cells, CD8+T cells, and Tregs were much more in VAV3low than in VAV3high groups (Supplementary Figure S1). In the NR3C2high group, the proportions of naïve B cells, memory resting CD4+T cells, resting NK cells, monocytes, M2, resting DCs, and resting Mast cells were higher, while the fractions of plasma cells, CD8+T cells, memory activated CD4+T cells, Th cells, γδT cells, and M0 macrophages were lower (Supplementary Figure S2).

To figure out the association between clinical characteristics and the two immune-related genes, we analyzed the mRNA expression levels of VAV3 and NR3C2 in different clinical subgroups. The classification standard was based on the previous literature (Meng et al., 2021b; Lou et al., 2021). The results showed that the expression of VAV3 had no significance between the elderly group (age ≤ 60 years) and young group (age > 60 years) (Supplementary Figures S3A,B). Besides, VAV3 expressed higher in females than in males. However, the expression level of VAV3 decreased with the increase of T stage, N stage, M stage, TNM stage, and G grades (Supplementary Figures S3C–G). It may meant that VAV3 was not only downregulated in ccRCC but also decreased as the malignancy of tumors increased. Similarly, the expression level of NR3C2 was not correlated with age (Supplementary Figure S4A) and gender (Supplementary Figure S4B) but importantly negatively related to T stage, N stage, M stage, TNM stage, and tumor G grade (Supplementary Figures S4C–G).

### Prediction of Potential Therapeutic Drugs

**FIGURE 9:**
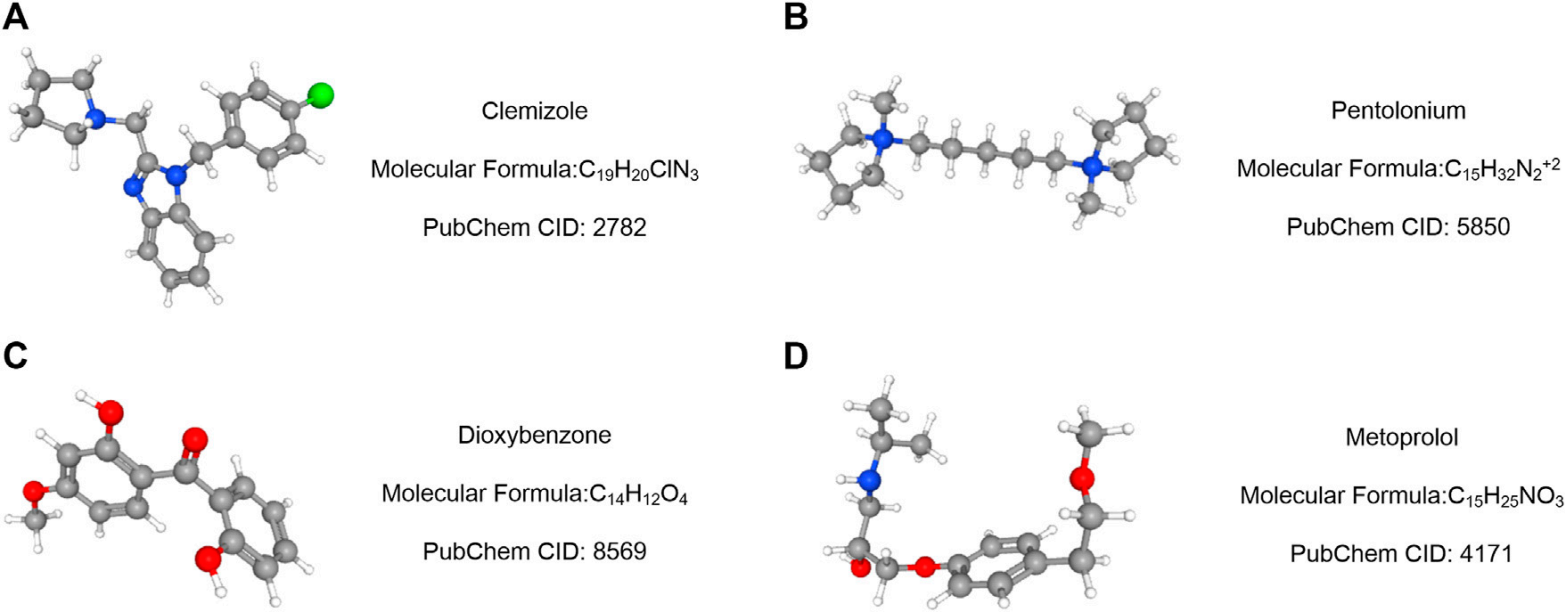
Drug prediction results based on three targeted genes (HAMP, NR3C2, and VAV3). **(A–D)** Important relative information of the potential drugs.

Using the Cmap database and three hub genes associated with hsa-miR-186-5p, we predicted potential drugs for ccRCC. NR3C2 and VAV3 were downregulated in ccRCC, while HAMP was upregulated. Drugs with negative connectivity scores were considered therapeutic. We finally selected five small molecular drugs in which enrichment < −0.7 and *p*-value < 0.01 (Figure 9; Table 2). Their structure and other information were presented through PubChem, in which Prestwick-691 could not be found.

**TABLE 2:** Potential drugs for treatment of ccRCC.

Cmap name	Mean	N	Enrichment	*p*-Value	Specificity	Percent non-null
Clemizole	−0.657	5	−0.773	0.00106	0	100
Pentolonium	−0.68	5	−0.764	0.00136	0	100
Dioxybenzone	−0.667	4	−0.784	0.00436	0.0054	100
Prestwick-691	−0.73	3	−0.865	0.00497	0.0395	100
Metoprolol	−0.677	4	−0.761	0.00662	0.0076	100

## Discussion

**FIGURE 10:**
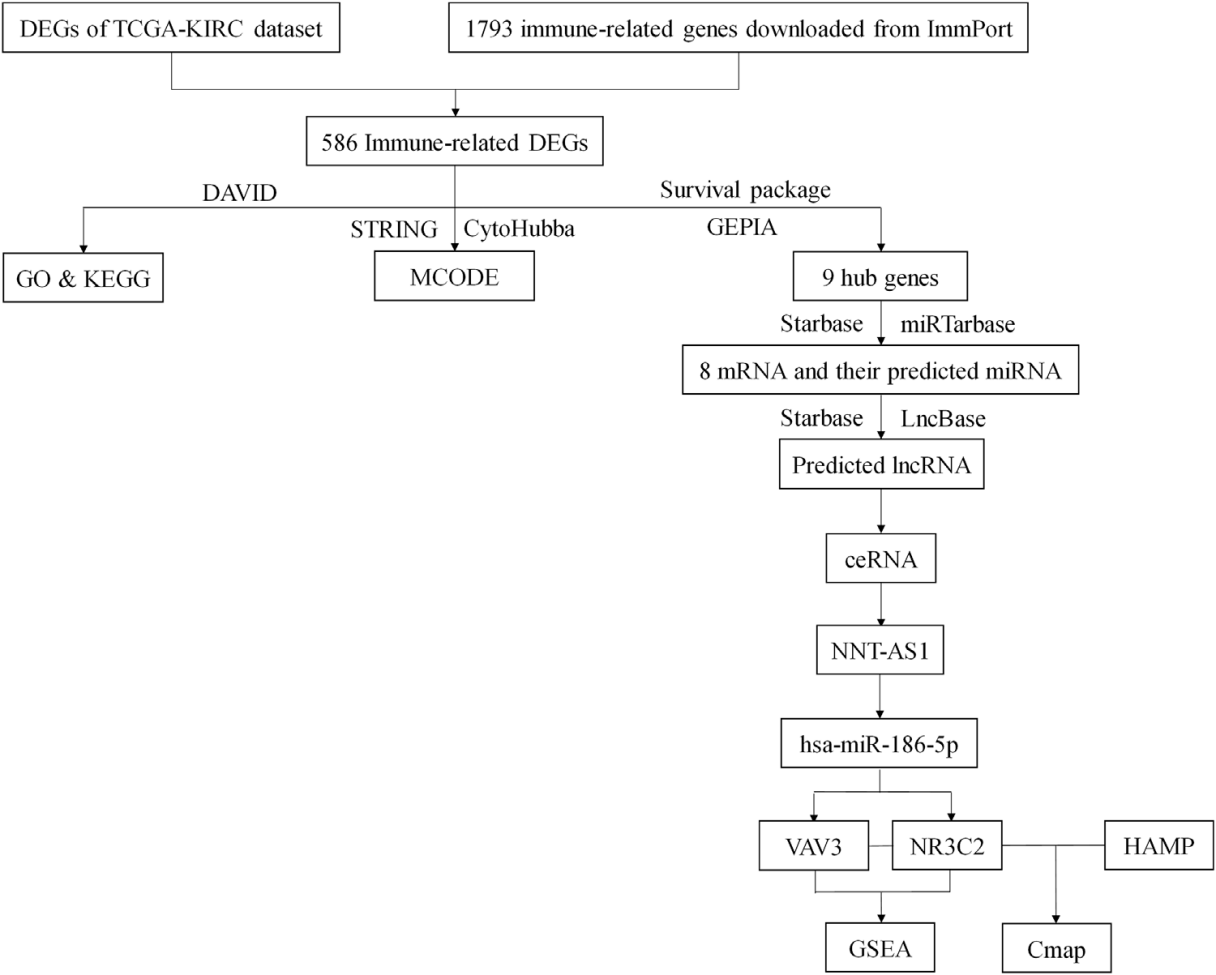
Flow chart of this study. DEGs, differentially expressed genes; TCGA, The Cancer Genome Atlas; KIRC, Kidney Clear Cell Carcinoma; DAVID, The Database for Annotation, Visualization, and Integrated Discovery; GO, Gene Ontology; KEGG, Kyoto Encyclopedia of Genes and Genomes.

Renal cancer is one of the top 10 cancers all over the world, and ccRCC makes up more than 75% of it. Although there had been much research studies to explore the pathogenesis and therapy for ccRCC, it still lacked suitable biomarkers and therapeutic targets. Since immunotherapy was considered a new hope for cancer therapy, we would like to figure out a novel immune-related ceRNA network that might serve as a prognostic marker and therapeutic entry point for ccRCC (Figure 10).

In this study, we took the intersection of DEGs of KIRC and 1793 immune genes to screen initially immune-related DEGs. Functional enrichment of all 586 DEGs was made, and we found they were associated with T-cell chemotaxis, B-cell chemotaxis, and multiple macrophage polarization-related signal pathways. The above biological processes and pathways were extensively studied and proved to be related to tumor development (Sousa et al., 2015; Chen et al., 2017; Choo et al., 2018; Locati et al., 2020). We further divided these genes into multiple modules and separately explored each modules’ function. Interestingly, many classic and important pathways were enriched in various modules at the same time. Both the NF-kappa B signaling pathway (Hagemann et al., 2008; Mann et al., 2017) and JAK-STAT signaling pathway (Jiménez-García et al., 2018; Irey et al., 2019) had been proved to be key pathways in macrophage polarization.

Survival analysis and multivariate cox regression analysis were used for screening hub genes with a prognostic value. Finally, nine genes (AGER, HAMP, LAT, LTB4R, NR3C2, SEMA3D, SEMA3G, SLC11A1, and VAV3) were determined by GEPIA further. GO enrichment results showed that four hub genes (AGER, HAMP, SLC11A1, and VAV3) were associated with macrophage activation, which had been proved before (Wyllie et al., 2002; Sindrilaru et al., 2009).

Then, we predicted miRNA according to the hub genes and online databases. The top 15 miRNA were calculated by the “CytoHubbe” plugin and selected for the following analysis. The expression level and prognostic value of the 15 miRNA were explored again. As a result, hsa-miR-186-5p was determined. Consistent with the ceRNA hypothesis, highly expressed hsa-miR-186-5p acted as an inhibitor to silence downstream genes, which meant that low-expressed genes (NR3C2 and VAV3) in ccRCC could be the target genes in the ceRNA. Next, we predicted lncRNAs for hsa-miR-186-5p using LncBase and Starbase. There were 29 lncRNAs in the intersection, while only NNT-AS1 perfectly satisfied multiple conditions including survival analysis, cox regression analysis, and ceRNA hypothesis. Finally, we constructed a new immune-related ceRNA network successfully, in which low-expressed NNT-AS1 downregulated NR3C2 and VAV3 to promote ccRCC through upregulating hsa-miR-186-5p. Although there had been no article proving the ceRNA network in ccRCC, the role of NNT-AS1 and the hsa-miR-186-5p axis had been explored in cervical cancer (Liu et al., 2020). The ceRNA network could also be associated with macrophage activation and polarization in the microenvironment. The Cmap database was used to predict potential drugs which might act on the ceRNA and treat ccRCC patients. Clemizole, pentolonium, dioxybenzone, and metoprolol are shown in Figure 9.

Our study had some limitations. We constructed a novel immune ceRNA network using various bioinformatics analyses and verified the expression of NNT-AS1, has-miR-186-5p, VAV3, and NR3C2. Although the correlation analysis results of above ceRNA components meet the ceRNA hypothesis, whether NNT-AS1 could truly downregulate hsa-miR-86-5p and further upregulate VAV3 and NR3C2 needed further experiment verification. Besides, the influence of the ceRNA network to the tumor microenvironment needed further investigation. We will explore the ceRNA network based on the article.

## Conclusion

In summary, we predicted a new ceRNA network for ccRCC, which could act as a prognostic biomarker and might contribute to the progression of ccRCC. Five potential drugs which might act on the ceRNA and treat ccRCC patients were predicted for future study.

## Data Availability

The original contributions presented in the study are included in the article/Supplementary Material; further inquiries can be directed to the corresponding author/s.
