# Supplementary material for: A Novel Immune-Related ceRNA Network and Relative Potential Therapeutic Drug Prediction in ccRCC
Source: Front Genet. 2022 Jan 25;12:755706. doi: 10.3389/fgene.2021.755706 (PMC8821820; doi:10.3389/fgene.2021.755706)
Supplement: Supplementary file 1 [file DataSheet1.doc]

A novel immune-related ceRNA network and relative potential therapeutic drugs prediction in ccRCC

Weiquan Li^1,2,3#^, Xiangui Meng^1,2,3#^, Hongwei Yuan^1,2,3#^, Wen Xiao^1,2,3*^, Xiaoping Zhang^1,2,3*^

^1^ Department of Urology, Union Hospital, Tongji Medical College, Huazhong University of Science and Technology, Wuhan 430022, China

^2^ Shenzhen Huazhong University of Science and Technology Research Institute, Shenzhen 518000, China

^3^ Institute of Urology, Tongji Medical College, Huazhong University of Science and Technology, Wuhan 430022, China

^#^XGM, WQL, and HWY contributed equally to this work

^*^*Correspondence to:*

Xiaoping Zhang, Department of Urology, Union Hospital, Tongji Medical College, Huazhong University of Science and Technology, 1277 Jiefang Avenue, Wuhan, Hubei Province, 430022(China). Phone: 86-18602752025; FAX number: 85776343; Email: [xzhang@hust.edu.cn](mailto:xzhang@hust.edu.cn).

Wen Xiao, Department of Urology, Union Hospital, Tongji Medical College, Huazhong University of Science and Technology, Wuhan, 430022, China; E-mail: xiaowenx11@163.com


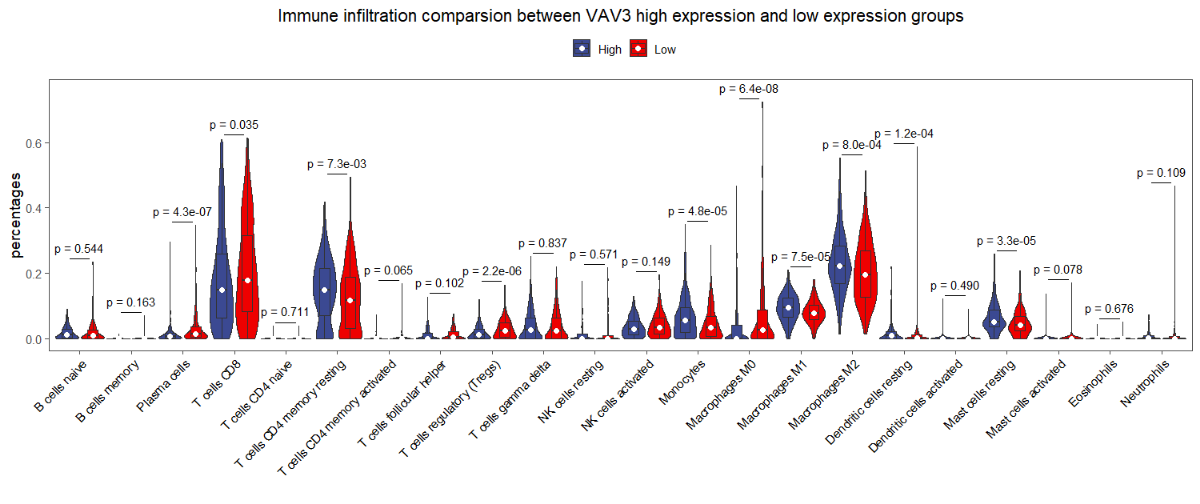
Figure S1. Immune infiltration analysis of VAV3 expression level. All 22 immune cells proportions were compared between VAV3high (blue) and VAV3low (red) groups.


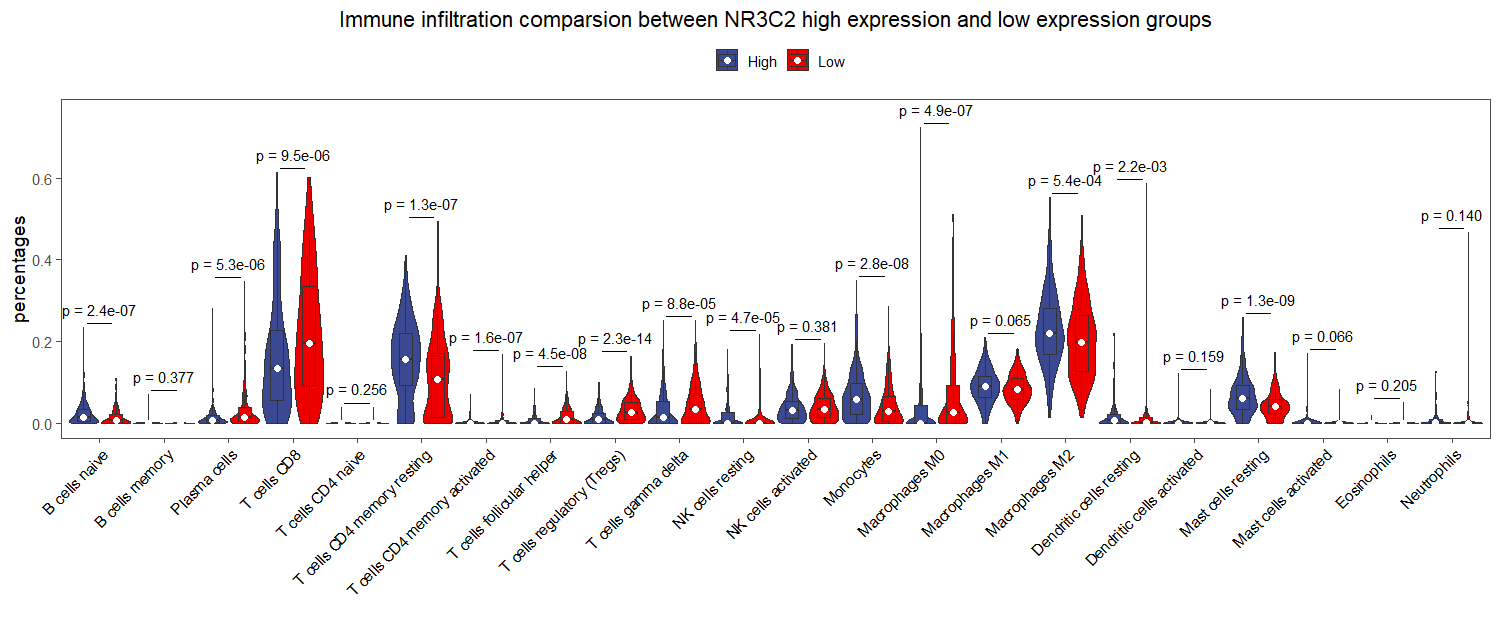


Figure S2. Immune infiltration analysis of NR3C2 expression level. All 22 immune cells proportions were compared between NR3C2high (blue) and NR3C2low (red) groups.


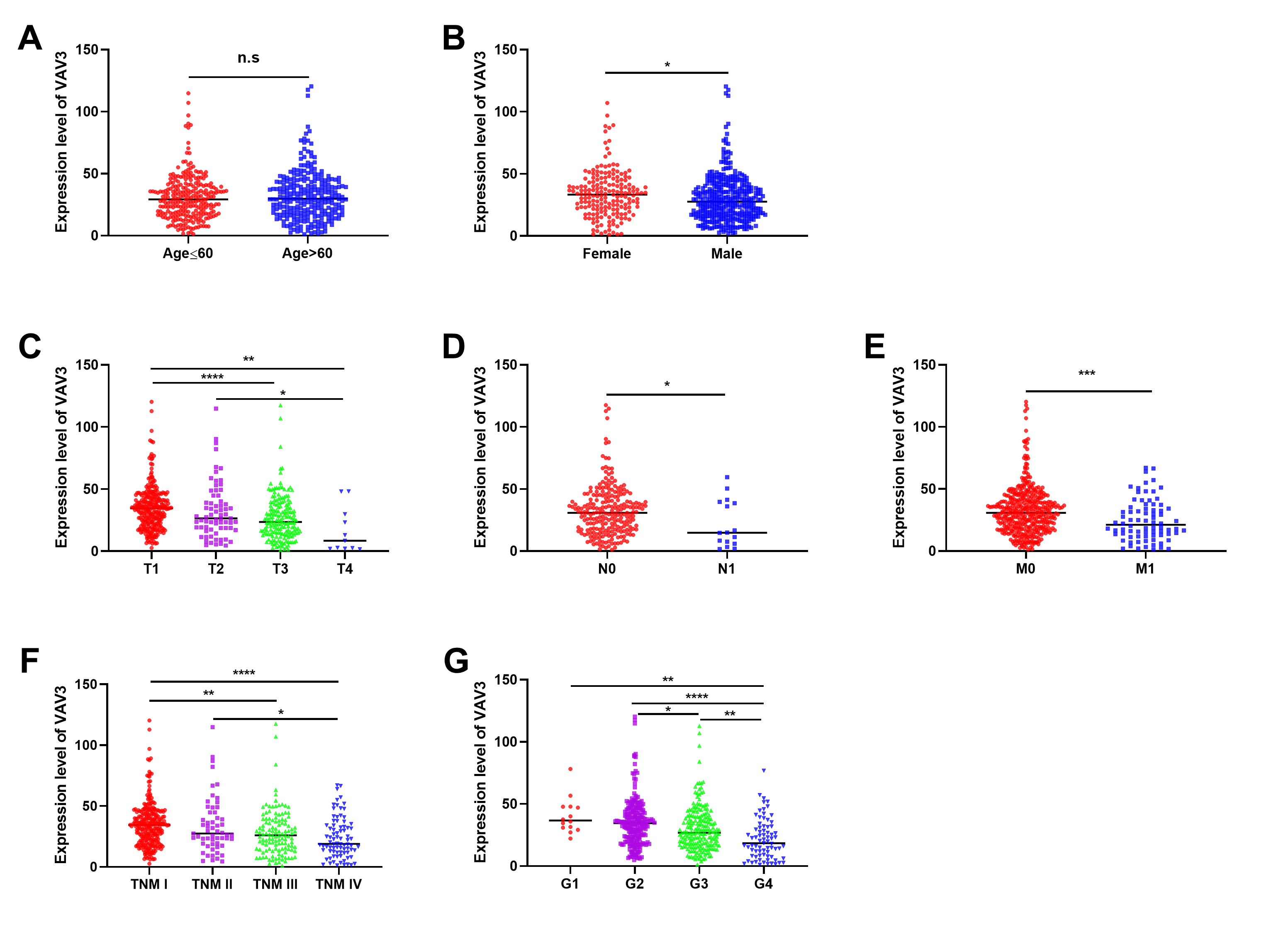


Figure S3. The association of VAV3 expression and different clinicopathological parameters. Correlation analysis of VAV3 with clinical characteristics were observed according to Age (A), Gender (B), T stage (C), N stage (D), M stage (E), TNM stage (F) and G grade (G). n.s means no significance; *p < .05; **p < .01; ***p < .001; ****p < .0001.


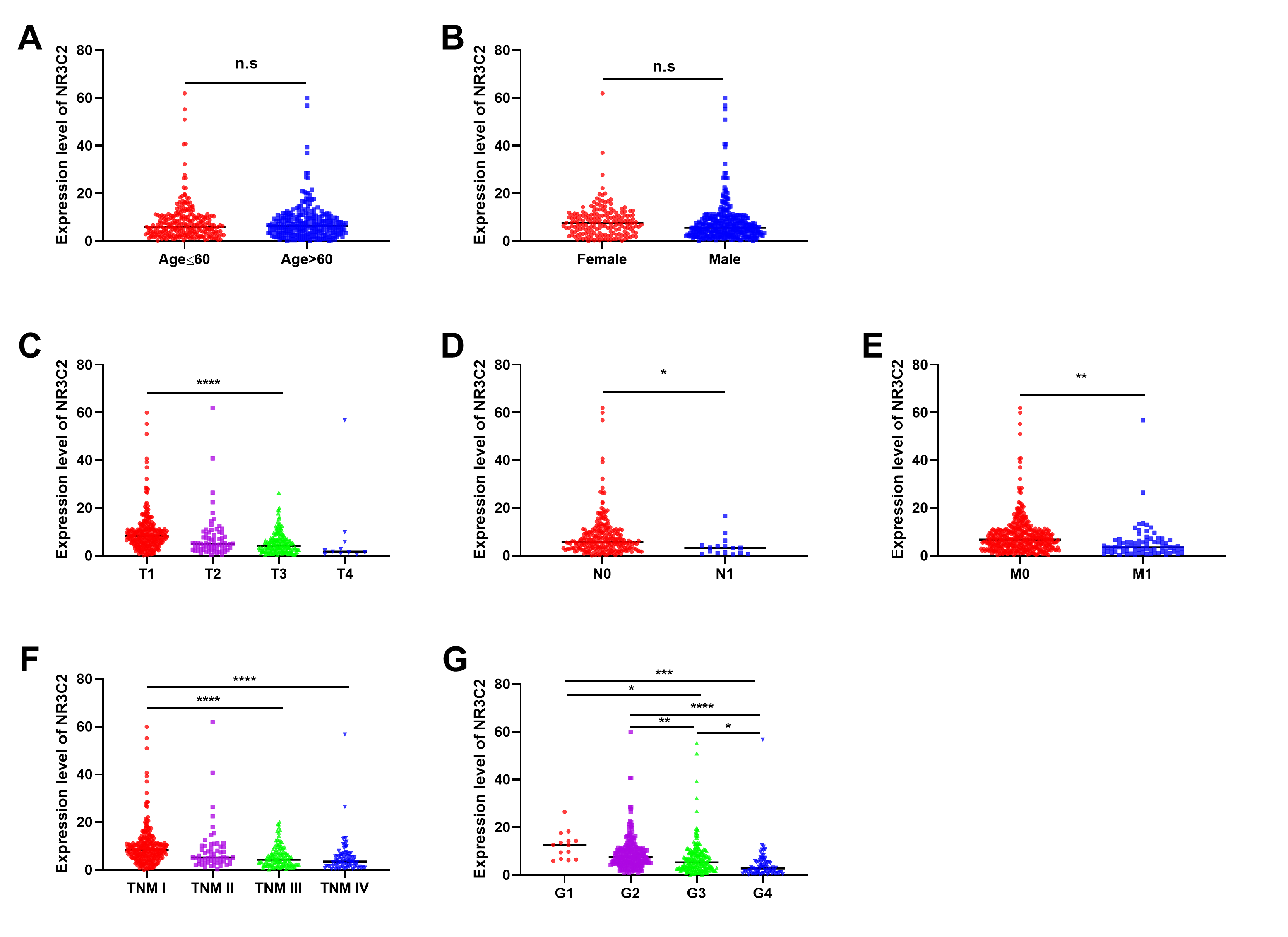


Figure S4. The association of NR3C2 expression and different clinicopathological parameters. Correlation analysis of VAV3 with clinical characteristics were observed according to Age (A), Gender (B), T stage (C), N stage (D), M stage (E), TNM stage (F) and G grade (G). n.s means no significance; *p < .05; **p < .01; ***p < .001; ****p < .0001.
